# Hyperosmotic stress stimulates autophagy via polycystin-2

**DOI:** 10.18632/oncotarget.18995

**Published:** 2017-07-05

**Authors:** Daniel Peña-Oyarzun, Rodrigo Troncoso, Catalina Kretschmar, Cecilia Hernando, Mauricio Budini, Eugenia Morselli, Sergio Lavandero, Alfredo Criollo

**Affiliations:** ^1^ Advanced Center for Chronic Diseases, Facultad Ciencias Quimicas y Farmaceuticas & Facultad Medicina, Universidad de Chile, Santiago, Chile; ^2^ Center for Molecular Studies of the Cell, Facultad de Medicina, Universidad de Chile, Santiago, Chile; ^3^ Instituto de Nutrición y Tecnología de los Alimentos, Universidad de Chile, Santiago, Chile; ^4^ Instituto de Investigación en Ciencias Odontológicas, Facultad de Odontología, Universidad de Chile, Santiago, Chile; ^5^ Departamento de Fisiología, Facultad de Ciencias Biológicas, Pontificia Universidad Católica de Chile, Santiago, Chile; ^6^ Department of Internal Medicine (Cardiology Division), University of Texas Southwestern Medical Center, Dallas, TX, USA

**Keywords:** hyperosmotic stress, polycystin-2, mTOR, Autophagy

## Abstract

Various intracellular mechanisms are activated in response to stress, leading to adaptation or death. Autophagy, an intracellular process that promotes lysosomal degradation of proteins, is an adaptive response to several types of stress. Osmotic stress occurs under both physiological and pathological conditions, provoking mechanical stress and activating various osmoadaptive mechanisms. Polycystin-2 (PC2), a membrane protein of the polycystin family, is a mechanical sensor capable of activating the cell signaling pathways required for cell adaptation and survival. Here we show that hyperosmotic stress provoked by treatment with hyperosmolar concentrations of sorbitol or mannitol induces autophagy in HeLa and HCT116 cell lines. In addition, we show that mTOR and AMPK, two stress sensor proteins involved modulating autophagy, are downregulated and upregulated, respectively, when cells are subjected to hyperosmotic stress. Finally, our findings show that PC2 is required to promote hyperosmotic stress-induced autophagy. Downregulation of PC2 prevents inhibition of hyperosmotic stress-induced mTOR pathway activation. In conclusion, our data provide new insight into the role of PC2 as a mechanosensor that modulates autophagy under hyperosmotic stress conditions.

## INTRODUCTION

Osmotic homeostasis is crucial for maintaining normal cell function. Under physiological conditions, renal tubule and gastrointestinal tract cells are routinely subjected to severe changes in osmolarity. However, in other cell types, hyperosmotic stress may promote various human pathologies. For instance, elevated hypertonicity in the extracellular fluid is often associated with ocular tissue disorders involving tear osmolarity and ocular surface inflammation [1, 2], diabetes [3], obesity [4], hypernatremia [5] and inflammatory bowel disease [6, 7]. Imbalanced fluid tonicity provokes osmotic stress, which triggers a series of adaptive mechanisms to ensure cell survival. Transporter translocation [8], transcription factor activation [9], osmolyte synthesis [10], upregulation of antioxidant and chaperone proteins [11], cytoskeletal remodeling [12] and cell volume changes [13] are activated rapidly during osmotic stress. Studies have also reported a correlation between osmotic stress and impaired cellular proteostasis [14–16]. Hyperosmotic stress leads to intracellular accumulation of damaged, aggregated, misfolded and oxidized proteins, which can activate cellular degradation mechanisms. Studies have shown that the main cellular protein turnover systems, the autophagy and ubiquitin proteasome pathways, are upregulated under hyperosmotic stress conditions [17, 18].

Macroautophagy, hereafter referred to as autophagy, is a catabolic pathway that is highly conserved from yeast to mammals [19]. During autophagy, the cargo, which consists of abnormal and aberrant proteins, proteins with short half-lives and aged or oxidized organelles, is sequestered into double-membrane vesicles known as autophagosomes [20]. Autophagosomes then fuse with the lysosomes to form autophagolysosomes, in which the cargo is degraded by hydrolytic enzymes [20]. Various autophagy-related (ATG) proteins are involved in initiation, nucleation and elongation of autophagosomes [21, 22]. In general, downregulation of ATG proteins strongly inhibits autophagy. The autophagic mechanism can be fine-tuned by the stress sensor proteins AMP-dependent kinase (AMPK) and the mechanistic target of rapamycin (mTOR) [20, 23]. Thus, under basal conditions, the constitutive Ser/Thr kinase activity of mTOR [23] represses autophagy. When cells are subjected to stressors such as nutrient deprivation, autophagy may be activated by suppression of mTOR activity or increased Ser/Thr kinase activity of AMPK [23].

Few studies have evaluated the role of hyperosmotic stress in autophagy induction. In yeast, osmolytes such as ammonium sulfate, raffinose and sorbitol are capable of inducing autophagy in a p38 mitogen-activated protein kinase (p38 MAPK)-dependent manner [24]. In rat notochordal cells, hyperosmotic solutions containing NaCl increase autophagy *via* AMPK. Similar results have been observed in Chinese hamster ovary (CHO) and porcine renal proximal tubule-like (LLC-PK) cell lines, in which hypertonic solutions of NaCl induce autophagosome formation [25–27]. Additionally, we have shown in previous studies that sorbitol-induced hyperosmotic stress activates nuclear factor kappa beta (NF-κB) in neonatal rat cardiomyocytes [28–30]. This transcription factor modulates the autophagic response in multiple cell types [28–30]. We have also reported that hyperosmolar solutions of sorbitol or mannitol lead to mitochondrial proton gradient dissipation, resulting in mitochondrial dysfunction [31]. This disruption may promote selective degradation of mitochondria by autophagy, a process known as mitophagy [17]. Although autophagy is recognized as an important adaptive response to hyperosmotic stress, few studies have explored autophagy in human cells, and little is known about the mechanotransducers involved in sensing osmotic changes and triggering autophagy.

Importantly, polycystins (PCs) represent a large family of proteins associated with mechanotransduction pathways [32, 33]. One member of this family, polycystin-2 (PC2), has been studied extensively due to its role in human polycystic kidney disease (PKD). PC2 forms a complex with polycystin-1 (PC1) that contributes to mechanosensation, detecting tonicity changes, fluid shear stress and fluid flow in the kidney. Specific PC2 mutations lead to PKD, which is characterized by impaired cell volume regulation leading to formation of kidney cysts [34]. Interestingly, rapamycin, a pharmacological inhibitor of mTOR and consequently a strong inducer of autophagy, attenuates PKD symptoms in patients and animal models by modulating cell size [35–38]. These findings are consistent with results in PC2-deficient cell lines, where correlations between PC and mTOR pathway activity have been found. PC2 and mTOR are involved in modulating stress-induced autophagy [39]. Collectively, these studies suggest that impaired autophagy may be related to altered PC2 activity [40–43]. Therefore, it is possible that PC2 is required not only for mechanosensation, as has largely been demonstrated, but also for adaptively maintaining proteostasis in cells subjected to osmotic stress.

Here, we show that osmotic stress, induced by treatment with hyperosmotic solutions of sorbitol or mannitol, induces autophagy in the human cell lines HeLa and HCT116. Moreover, we show that hyperosmotic stress induces autophagy by modulating the classic mTOR and AMPK pathways. Finally, we show that PC2 is required for hyperosmotic stress-induced autophagy, produced by modulation of the mTOR pathway.

## RESULTS

### Hyperosmotic stress induces autophagy

**Figure 1:**
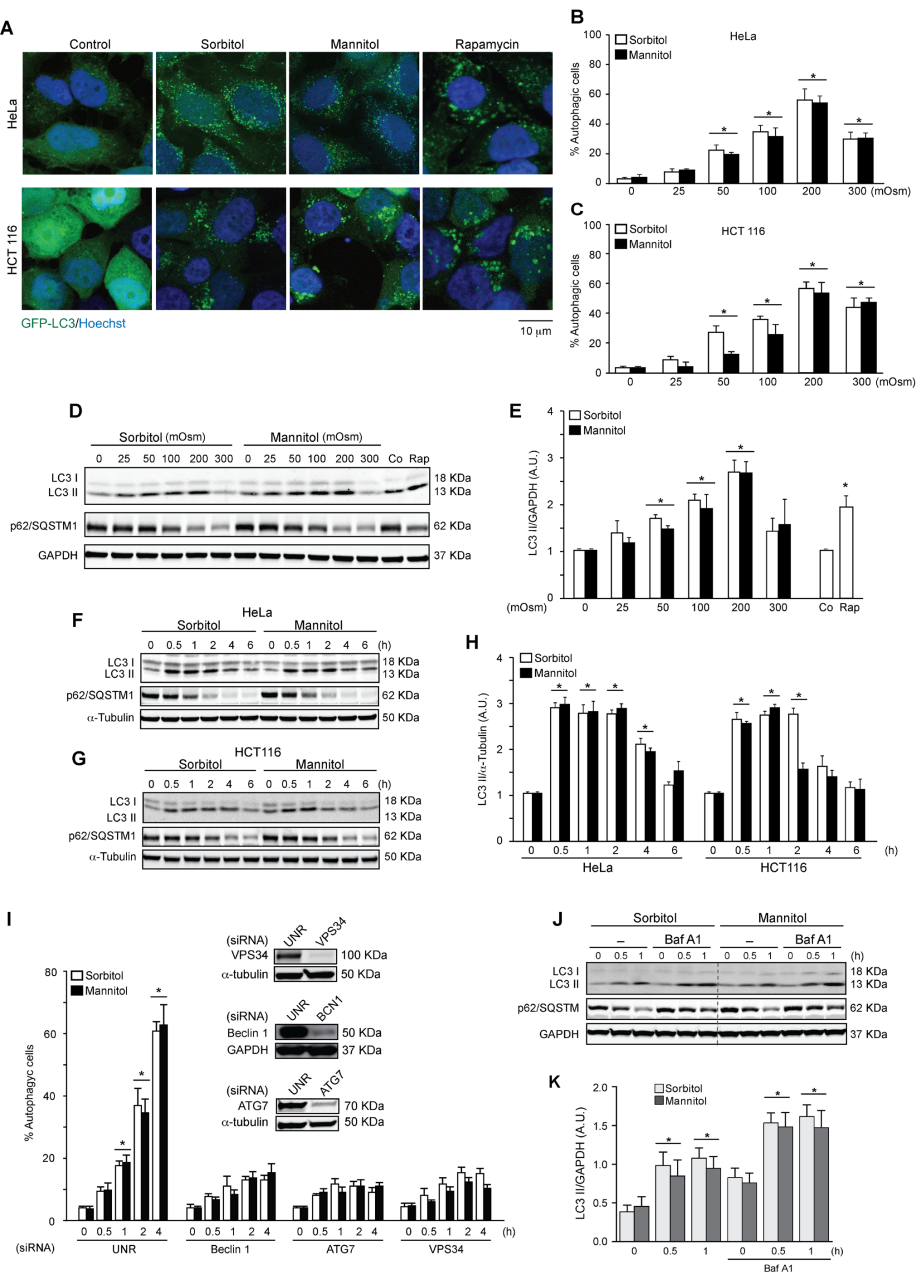
Hyperosmotic stress stimulates autophagy HeLa and HCT 116 cells were transduced with an adenovirus coding for GFP-LC3 (Ad GFP-LC3) for 24 h. Cultures were then exposed to different concentrations (25-300 mOsm) of sorbitol or mannitol for 2 h. Subsequently, autophagy was evaluated by fluorescent microscopy. 1 μM of rapamycin was used as a positive control to induce autophagy. Nuclei were dyed with 10 ng/mL of DAPI. Representative pictures are shown in **A.**. The percentage of cells with GFP-LC3 puncta (autophagic cells) in HeLa and HCT116 are shown in **B.** and **C.**, respectively (mean ± SEM, *n* = 3, *p < 0.05 *vs*. 0 mOsm). LC3 I-to-LC3 II conversion and p62/SQSTM1 depletion were assessed by Western blot analysis in HeLa and HCT116 cells treated with various concentrations of sorbitol or mannitol (25-300 mOsm) **D.**, **E.** for the indicated times (0.5-6 h) **F.**-**H.** Representative gels are shown in **D.**, **F.** and **G.**. Quantification of gel bands is shown in **E.** and **H.** (mean ± SEM, *n* = 3, *p < 0.05 *vs*. 0 mOsm or 0 h). HeLa cells were transfected with specific siRNAs against Beclin 1, ATG7 or VPS34, followed by infection with Ad GFP-LC3. Subsequently, cells were treated with 200 mOsm of sorbitol or mannitol for 0, 0.5, 1, 2 or 4 h. Autophagy was evaluated by fluorescent microscopy. The percentage of autophagic cells was quantified, shown in **I.** (*p < 0.05 *vs*. 0 h) and representative gels inserted in the graphic indicate the efficiency of siRNA downregulation for Beclin 1, ATG7 and VPS34 **I.** An unrelated siRNA (UNR) was used as a control. GAPDH and α-tubulin were used as loading controls **J.**-**K.** The effect of BafA1 treatment on LC3 I-to-LC3 II conversion and p62/SQSTM1 degradation under hyperosmotic stress conditions was assessed. HeLa cell cultures were exposed to sorbitol or mannitol (200 mOsm) for 0, 0.5 or 1 h in the presence or absence of 50 nM of BafA1. LC3 II and p62/SQSTM1 levels were then determined by Western blot analysis **J.**. GAPDH levels were monitored as a loading control. Quantification of gel bands is shown in **K.** (mean ± SEM, *n* = 3, *p < 0.05 *vs*. 0 h).

To evaluate the effect of hyperosmotic stress on autophagy, two different human cell lines (the human colon tumor cell line HCT116 and the human cervical cancer cell line HeLa) were exposed to various concentrations of sorbitol and mannitol, two osmolytes widely used to increase tonicity in cells [10]. Both compounds induced autophagy at different concentrations, reaching the highest levels at 200 mOsm, as assessed by quantification of autophagic vacuoles with fluorescence microscopy (Figures 1A-1C) and quantification of LC3 I-to-LC3 II conversion by Western blot analysis (Figures 1D, 1E). Time-dependent changes in autophagy were also assessed in HeLa and HCT116 cells treated with sorbitol or mannitol. Increased autophagy was observed at 0.5, 1 and 2 h post-stimulation with sorbitol or mannitol (200 mOsm) (Figures 1F-1H). We also analyzed levels of p62/SQSTM1, an autophagosome cargo protein that is specifically degraded when autophagy is upregulated [44]. p62/SQSTM1 was degraded when cells were treated with sorbitol or mannitol at 200 or 300 mOsm (Figure 1D) for 2, 4 or 6 h (Figures 1F, 1G), confirming autophagy induction. Importantly, all experiments were also performed with rapamycin as a positive control. Rapamycin is a classic autophagy inducer and, as predicted, stimulated LC3 I-to-LC3 II conversion and p62/SQSTM1 degradation in HeLa cells [45, 46] (Figures 1D, 1E).

To confirm that hyperosmotic stress induces autophagy, we inhibited the autophagic pathway using siRNAs against classic components of the autophagic machinery, such as Beclin 1, ATG7 and vacuolar protein sorting 34 (VPS34) [47–49]. Beclin 1 and VPS34 are part of the class III phosphatidylinositol 3-kinase complex (PI3KC3), which is implicated in the formation of new autophagosomes. ATG7 is an E1-like activating enzyme involved in the ubiquitin-like systems required for autophagosome elongation [20–22]. Downregulation of Beclin 1, ATG7 and VPS34 blocked autophagosome formation in HeLa cells treated with sorbitol or mannitol (Figure 1I). Taken together, these data suggest that hyperosmotic stress induces autophagy.

Elevated autophagosome levels might be caused by increased formation of new autophagosomes or by decreased autophagosome degradation. To discriminate between these two options, autophagic flux was evaluated. This term refers to the entire dynamic process of autophagy from autophagosome synthesis to degradation within the lysosomes [44, 50]. We exposed HeLa cells to sorbitol or mannitol (200 mOsm) for 0.5 or 1 h in the presence or absence of 50 nM of Bafilomycin A1 (BafA1). BafA1 is a classical inhibitor of autophagic flux as it blocks the vacuolar H^+^ATPase, preventing autophagosome-lysosome fusion [44, 51]. The LC3 II and p62/SQSTM1 levels measured by Western blot analysis indicated that treatment with BafA1 enhanced sorbitol- and mannitol-induced autophagy (Figures 1J, 1K). Collectively, these results indicate that various stimuli that promote hyperosmotic stress also induce autophagic flux in HeLa cells.

### Hyperosmotic stress inhibits mTOR while activating the AMPK pathway

**Figure 2:**
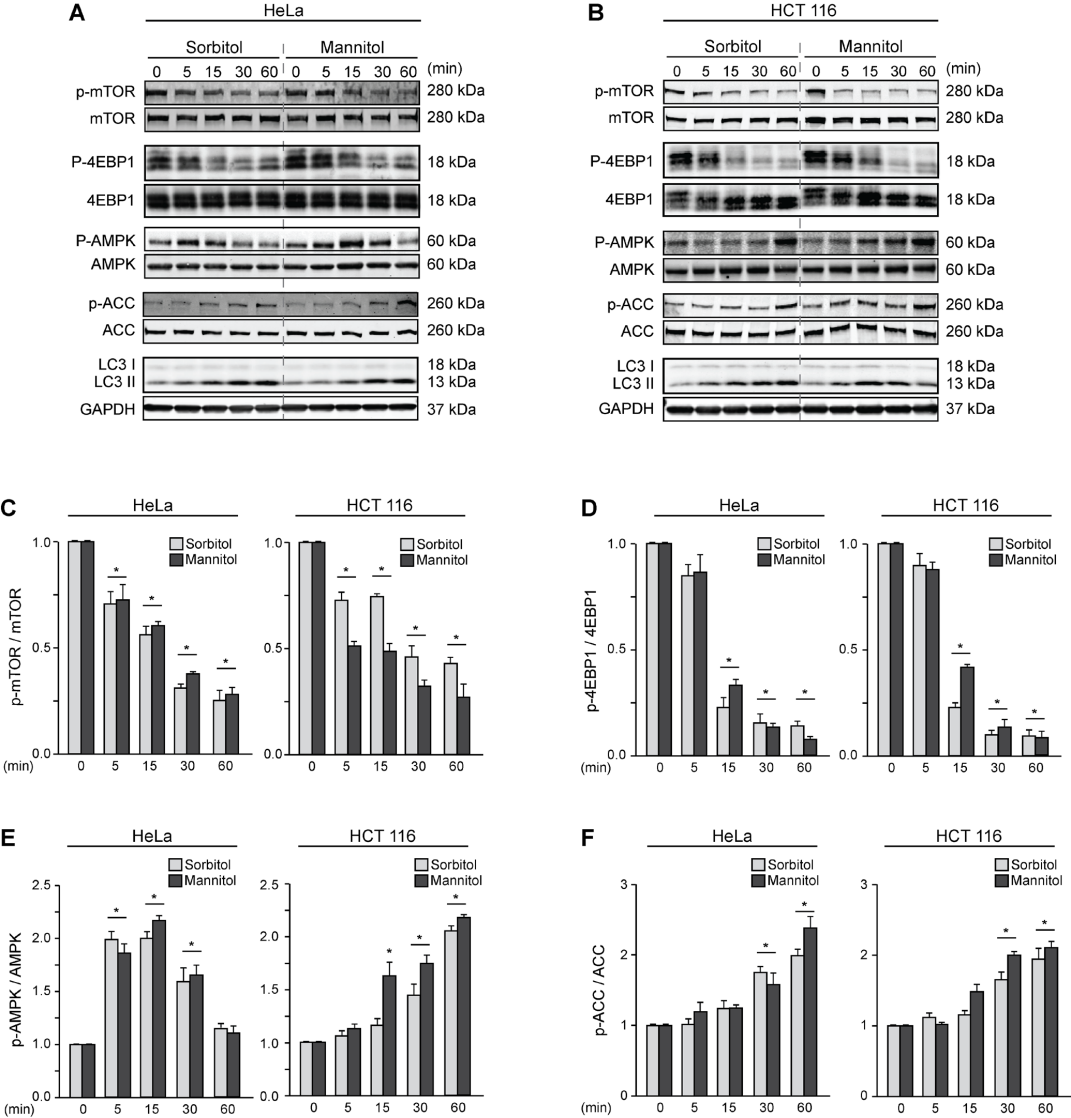
Hyperosmotic stress modulates the mTOR and AMPK pathways HeLa and HCT116 cultures were exposed to sorbitol or mannitol (200 mOsm) for 0, 5, 15, 30 or 60 min. Total and phosphorylated forms of mTOR, 4EBP1, AMPK, ACC and LC3 I-to-LC3 II conversion were evaluated by Western blot analysis. Representative gels are shown in **A.** and **B.**. GAPDH levels were monitored as a loading control. Quantification of gel bands is shown in **C.**-**F.** (mean ± SEM, *n* = 3, *p < 0.05 *vs*. 0 min).

We then evaluated the effect of sorbitol- and mannitol-induced autophagy on kinases that regulate the autophagic pathway. To this end, we determined the phosphorylation status of mTOR, which suppresses autophagy [23]; AMPK, which induces autophagy; and their downstream effectors, eukaryotic translation initiation factor 4E-binding protein 1 (4EBP1) and acetyl-CoA carboxylase (ACC), respectively [52]. HeLa and HCT116 cells were treated with sorbitol and mannitol (200 mOsm) for 0, 5, 15, 30 or 60 min (Figures 2A-2F). mTOR phosphorylation decreased significantly in both cell types after only 5 min of treatment with sorbitol or mannitol (Figures 2A-2C). 4EBP1 phosphorylation also decreased significantly after 15 min of treatment (Figures 2A, 2B, 2D). Accordingly, phosphorylation of AMPK and its downstream effector ACC increased significantly (Figures 2A, 2B, 2E, 2F). Importantly, we confirmed that autophagy also increased significantly at the same time points, as measured by the conversion of LC3 I to LC3 II (Figures 2A, 2B). Taken together, these experiments indicate that the classical pathways involved in the control of autophagy are affected by hyperosmotic stress.

### PC2 is required for hyperosmotic stress-induced autophagy

**Figure 3:**
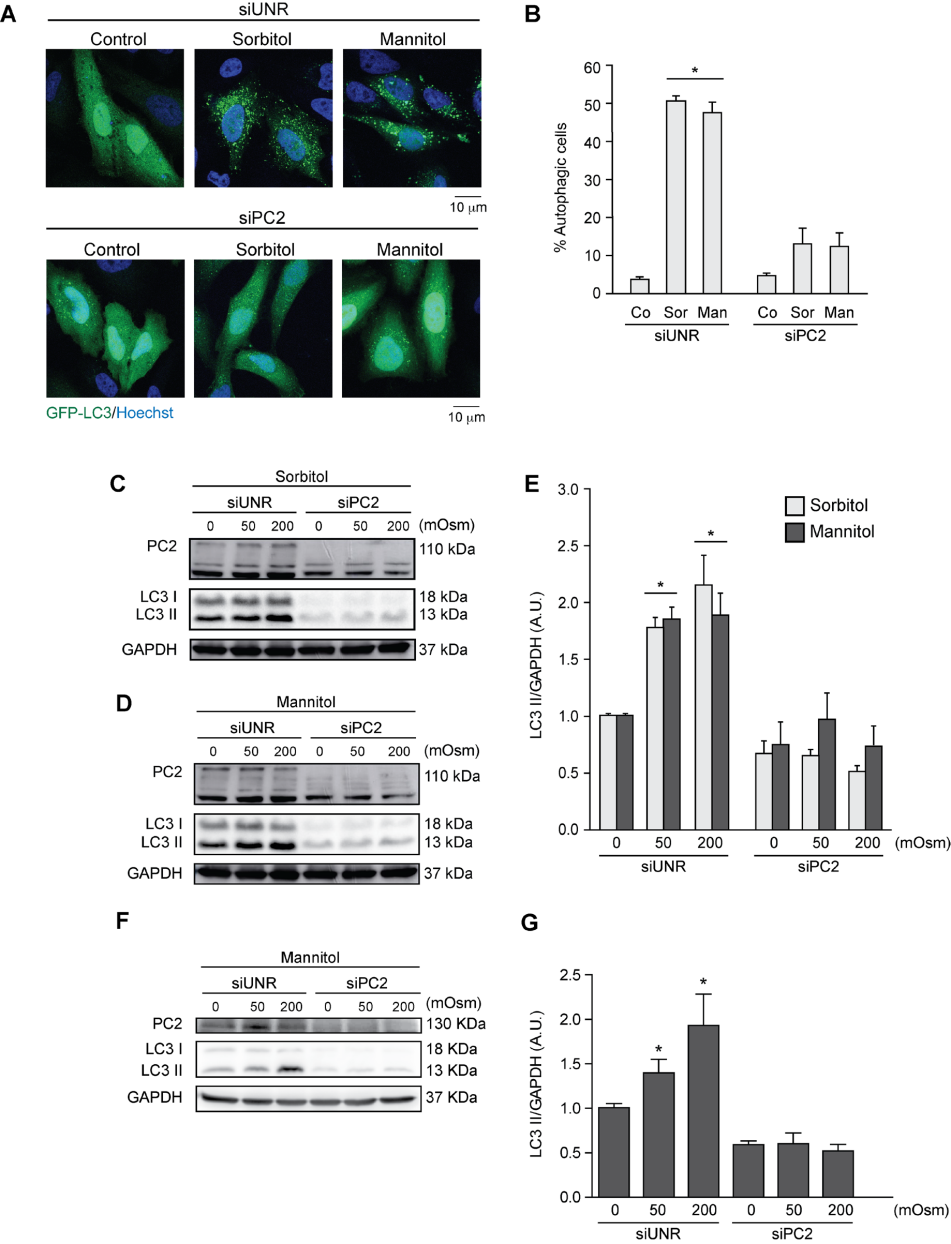
PC2 is required for hyperosmotic stress-induced autophagy PC2 was downregulated in HeLa cells by a specific siRNA against PC2. An unrelated siRNA (UNR) was used as a control. Subsequently, cells were infected with AdGFP-LC3 for 24 h and treated with sorbitol or mannitol (200 mOsm) for 2 h. Cells were fixed, and autophagy was evaluated by fluorescence microscopy. Representative pictures are shown in **A.** The percentage of autophagic cells is shown in **B.** (mean ± SEM, *n* = 3, *p < 0.05 *vs*. Co siUNR). Nuclei were dyed with 10 ng/mL of DAPI **C.**-**G.** PC2 was downregulated in HeLa **C.**-**E.** and HCT116 **F.**-**G.** cells with a specific siRNA against PC2. LC3 I-to-LC3 II conversion was evaluated by Western blot analysis in HeLa **C.**-**E.** and HCT116 **F.**-**G.** cells exposed to sorbitol or mannitol (0, 50 or 200 mOsm) for 2 h. Representative gels are shown in **C.**, **D.** and **F.**. Quantification of gel bands is shown in shown in **E.** and **G.** (mean ± SEM, *n* = 3, *p < 0.05 *vs*. 0 mOsm siUNR). GAPDH levels were used as a loading control.

PC2 is a member of the PC protein family, which localizes to membranous compartments such as the cytoplasm, endoplasmic reticulum and primary cilium membranes [53–55]. Studies have demonstrated that PC2 participates in mechanotransduction mechanisms, especially in the kidney cells, which are constantly subjected to fluctuations in osmolarity [33, 53, 56]. Since hyperosmotic stress generates mechanical stress by altering cell volume and cytoplasm membrane tension [57, 58], we evaluated the role of PC2 in hyperosmotic stress-induced autophagy. HeLa cells were exposed to sorbitol or mannitol (50 or 200 mOsm) after transfection with an unrelated siRNA (siUNR) or siRNA against PC2 (siPC2). We assessed autophagy by counting the percentage of cells with autophagic puncta with fluorescence microscopy (Figures 3A, 3B) and by quantifying LC3 I and LC3 II levels by Western blotting (Figures 3C-3E). The data, obtained using the two different approaches, show that downregulation of PC2 inhibited hyperosmotic-stress induced autophagy. These results were confirmed in HCT116 cells (Figures 3F, 3G). Given that decreased LC3 I levels are observed under control conditions when PC2 is downregulated, we evaluated the effect of siRNA against PC2 on *MAP1LC3B* gene expression, using real-time quantitative reverse transcription (RT-qPCR). Our results showed that *MAP1LC3B* expression was unaltered by downregulation of PC2, indicating that regulation of PC2-mediated autophagy is independent of *MAP1LC3B* expression (Supplementary Figure S1).

**Figure 4:**
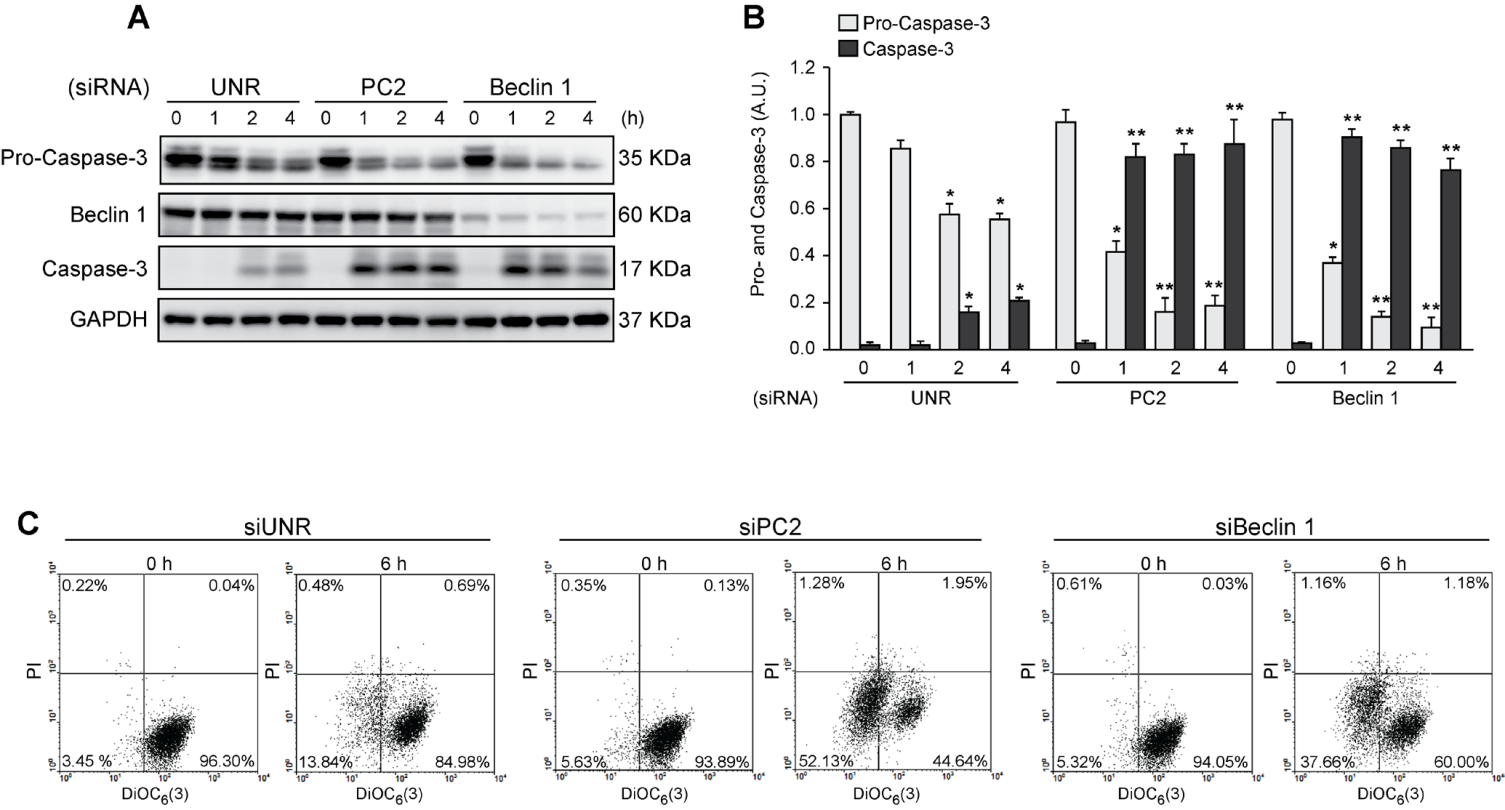
Pro-survival role of autophagy in cells subjected to hyperosmotic stress HeLa cells were transfected with an unrelated control siRNA (siUNR) or specific siRNAs against PC2 and Beclin 1. 48 h later, cells were exposed to sorbitol (200 mOsm) at the indicated times. Pro-caspase-3, Beclin 1 and caspase-3 levels were evaluated by Western blot analysis. GAPDH levels were used as a loading control. Representative gels are shown in **A.** Quantification of gel bands is shown in **B.** (mean ± SEM, *n* = 3, *p < 0.05 *vs*. 0 h siUNR, **p < 0.01 *vs*. 0 h siUNR). Alternatively, cells were submitted to cytofluorimetric analysis of mitochondrial membrane potential (DiOC_6_(3) staining) and viability (PI staining). **C.** Representative dot plots of HeLa cells treated for 6 h with 200 mM sorbitol are shown (*n* = 3).

Autophagy is considered to be a pro-survival mechanism that prevents cell death under stress conditions [59]. When autophagy was blocked by downregulation of Beclin 1, an essential autophagic protein [47], exposing HeLa cells to sorbitol enhanced apoptosis, as indicated by elevated short caspase-3 levels (Figures 4A, 4B). Interestingly, similar findings were observed when PC2 was downregulated. These data were also consistent with the results for mitochondrial transmembrane potential (∆Ψ_m_), evaluated by cytometry with the ∆Ψ_m_-sensitive dye DiOC_6_(3). Given that dissipation of ∆Ψ_m_ is considered a “point of no return” in the apoptosis pathway [60, 61], these findings indicate that HeLa cells subjected to hyperosmotic stress were more sensitive to apoptosis when PC2 was downregulated (Figure 4C). Collectively, these results support previous findings suggesting that PC2 is required for hyperosmotic stress-induced autophagy.

### PC2 is required for hyperosmotic stress-induced downregulation of the mTOR pathway

**Figure 5:**
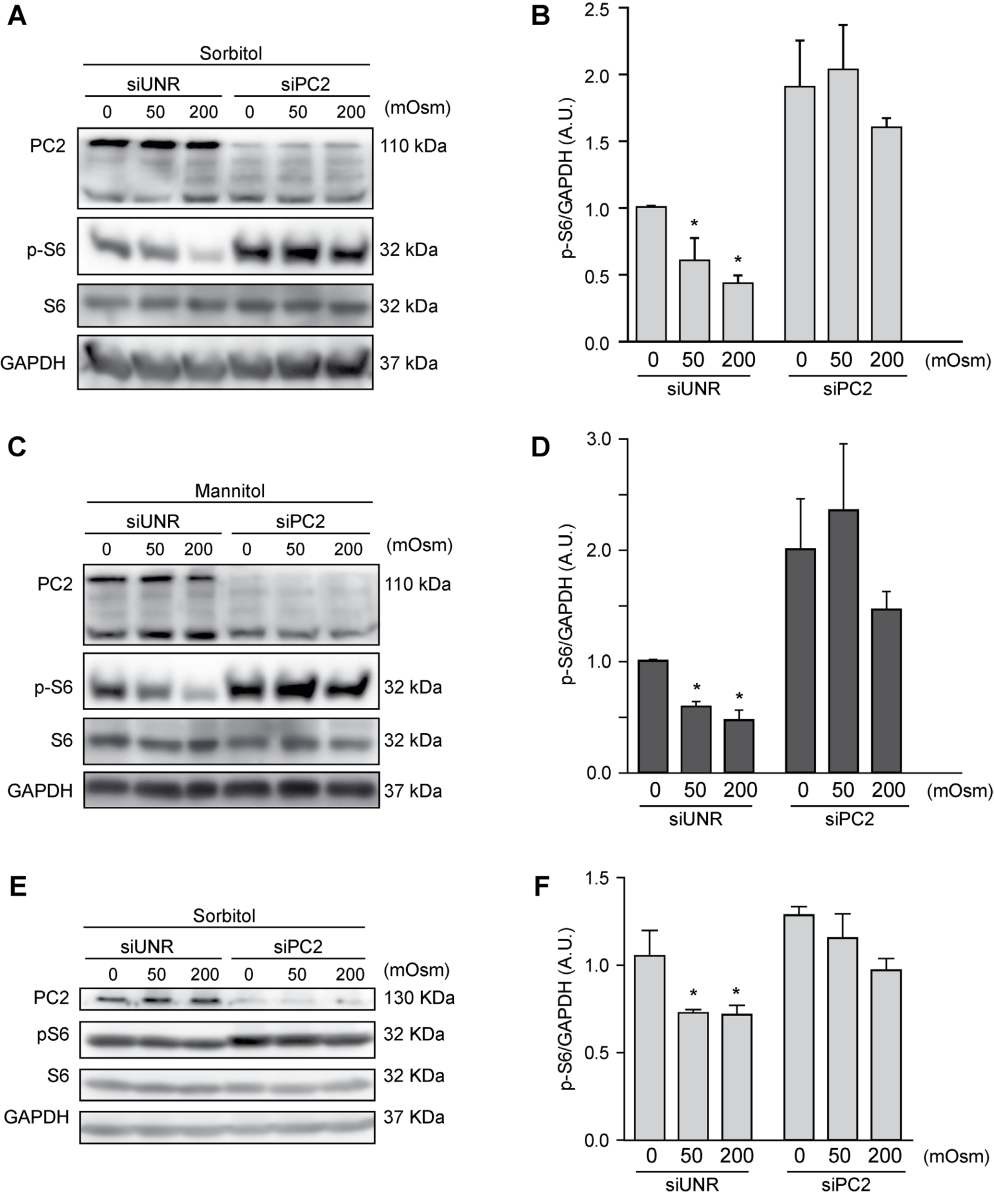
PC2 modulates the mTOR pathway under hyperosmotic stress conditions PC2 was downregulated in HeLa **A.**-**D.** and HCT116 **E.**-**F.** cells using a specific siRNA against PC2. An unrelated siRNA (UNR) was used as a control. Subsequently, cells were treated with 0, 50 or 200 mOsm sorbitol or mannitol and p-S6, S6 and PC2 levels were evaluated by Western blot analysis. Representative gels are shown in **A.**, **C.** and **E.**. Quantification of gel bands is shown in **B.**, **D.** and **F.** (mean ± SEM, *n* = 3, *p < 0.05 *vs*. 0 mOsm siUNR). GAPDH levels were used as a loading control.

Our previous data show that the mTOR pathway is modulated by hyperosmotic stress (Figures 2A-2D). Because mTOR, as previously mentioned, is a classic autophagic regulator, we evaluated whether PC2 affects the mTOR pathway under hyperosmotic stress conditions. To this end, we treated HeLa cells with sorbitol or mannitol (50 or 200 mOsm) for 30 min in the presence of PC2 or after siRNA-mediated downregulation of PC2. Our results show that the absence of PC2 prevented a decrease in S6 phosphorylation. This protein is downstream of mTOR, and S6 phosphorylation typically decreases when autophagy is induced [23]. Therefore, PC2 may be required for hyperosmotic-stress-induced mTOR pathway downregulation (Figures 5A-5D). Similar results were found in HCT116 cells, suggesting this mechanism occurs in different cell types (Figures 5E-5F). These data indicate that PC2 may regulate hyperosmotic stress-induced autophagy by modulating the mTOR pathway.

## DISCUSSION

Tissues in our body are constantly exposed to systemic and local osmotic alterations [62]. Severe osmotic changes in extracellular fluids are observed under both physiological and pathological conditions. Various mechanisms serve to maintain osmotic homeostasis in cells. While epithelial cells in the renal tubules or duodenal cells in the intestinal tract are routinely subject to severe osmotic variations under physiological conditions [56, 63], other cell types cannot cope with the osmotic variations that characterize some pathologies [64]. Unbalanced osmolarity is associated with disorders such as obesity [4], eye diseases [1], bowel diseases [6], cardiovascular disorders [65, 66], liver diseases [67] and cystic fibrosis [68, 69], among others [64]. *In vitro* experiments have demonstrated that hyperosmotic conditions stimulate rapid osmoadaptive responses in cells, including synthesis and transport of osmolytes, upregulation of antioxidant pathways and stimulation of mechanisms that promote cell volume recovery [70]. Importantly, we and others demonstrated that a failure of the osmoprotective mechanisms under hyperosmotic stress conditions leads apoptosis [66, 71–73].

Autophagy, along with the unfolded protein response and the ubiquitin-mediated protein degradation pathway, is a cellular mechanism involved in protein homeostasis [17, 18, 74]. Studies have shown that hyperosmotic stress induces autophagy [25–27]. For example, exposing cells to a hyperosmolar solution of NaCl induces autophagy in Chinese hamster ovary (rCHO) [26], porcine renal proximal tubule-like LLC-PK1 [27] and rat notochordal cells [25]. Despite these findings, there was no evidence available to date to confirm whether hyperosmotic stress induces autophagy in human cells. In the present study, we exposed human cells to sorbitol and mannitol, which belong to the carbohydrate osmolyte polyol family [10]. In mammals, sorbitol is the most predominant osmoprotective carbohydrate compound. Mannitol, which is considered chemically to be a sorbitol isomer, is widely used to modify tonicity in biological fluids [10]. Several studies have used NaCl to study cellular osmoadaptive mechanisms *in vitro*. However, NaCl also activates and increases Na^+^/K^+^-ATPase expression, whereas mannitol does not have this effect [75]. Therefore, we used osmolytes whose effects are limited to their osmotic capacity and do not affect ionic conductivity or transport across the membrane [76, 77].

This study shows that hyperosmotic stress stimulates autophagy in human cell lines (HeLa and HCT116), as indicated by relocalization of LC3 in autophagy vacuoles, LC3 I-to-LC3 II conversion and p62/SQSTM1 degradation (Figure 1). Additionally, specific knockdown of essential autophagy genes such as Beclin 1, ATG7 and VPS34 blunted hyperosmotic stress-induced autophagy (Figure 1). Autophagy flux experiments using HeLa cells in the presence and absence of BafA1 were also consistent with the previous results, demonstrating that both sorbitol and mannitol increased autophagic flux (Figure 1).

While AMPK induces autophagy by phosphorylating the positive regulator of autophagy unc-51-like kinase 1 protein (ULK1) on Ser317 and Ser77 [78], mTOR suppresses autophagy by phosphorylating on Ser757, Ser758 and Ser638 [79]. However, the classical AMPK- and mTOR-dependent autophagy does not always occur [23, 80]. Our results show that the AMPK and mTOR pathways were up- or downregulated, respectively, when HeLa and HCT116 cells were treated with sorbitol or mannitol, revealing that classical regulatory mechanisms are involved in modulating autophagy under hyperosmotic stress conditions (Figure 2).

PC2 is a mechanosensor required for activation of pro-survival cell signals, especially in renal tubules where cells are subject to physiological fluid flow [33]. A recent study showed that fluid shear stress induces autophagy *in vitro*, mediated by the primary cilium and ciliary PC2 through an mTOR-dependent mechanism [39]. Consistently, we found here that PC2 was required for hyperosmotic stress induced-autophagy in HeLa and HCT116 cells as evaluated by relocalization of GFP-LC3 in autophagy vacuoles and LC3 I-to-LC3 II conversion (Figure 3). Additionally, our data showed that downregulation of PC2 or Beclin 1 not only inhibited autophagy, but also enhanced levels of the effector short caspase-3, consistent with increased ∆Ψ_m_ dissipation (Figure 4). This finding suggests that PC2-mediated autophagy is an adaptive mechanism that prevents cell death under hyperosmotic stress conditions.

Given that our results showed that the mTOR pathway was inhibited under hyperosmotic stress conditions and that PC2 is required for hyperosmotic stress-induced autophagy, we evaluated whether PC2 is implicated in mTOR inhibition under hyperosmotic conditions. Our findings showed that downregulation of PC2 prevents the mTOR pathway inhibition induced by hyperosmotic stress (Figure 5). These data suggest that PC2 is required to induce autophagy *via* the mTOR pathway. Collectively, these data provide new insights into the role of PC2 in regulating autophagy in human cells under conditions of hyperosmotic stress.

## MATERIALS AND METHODS

### Cell culture and treatment

Human cervical cancer cell line (HeLa) cells from ATCC were grown in DMEM (glucose, 4.5 g/L) containing L-glutamine and 110 mg/L sodium pyruvate, supplemented with 10% FBS and 10 mM HEPES buffer. Human colon cancer cell line (HCT116) cells were maintained in McCoy's 5A medium (glucose 4.5 g/L) supplemented with 10% FBS and 10 mM HEPES buffer. All media, supplements and reagents for cell culture were purchased from Gibco, Invitrogen (Carlsbad, CA, USA). Cells were treated with D-sorbitol or D-mannitol (Sigma-Aldrich Corporation, St. Louis, MO, USA) at concentrations from 0 to 300 mOsm in the presence or absence of Bafilomycin-A1 (50 nM), for the times indicated in the various experiments. All experiments were independently repeated at least three times.

### siRNA transfection and adenovirus infection

siRNAs were purchased from the Sigma-Aldrich Corporation. An unrelated siRNA sequence was used as a negative control. Lipofectamine RNAiMax (Invitrogen) and Opti-MEM culture medium were used for siRNA transfection. 36 h after transfection, cells were stimulated, and the target protein was measured to evaluate the efficiency of the various siRNAs. For adenovirus-mediated protein overexpression, cells were incubated for 12 h with the AdGFP-LC3 adenovirus, followed by siRNA transfection and/or treatment with mannitol or sorbitol for the indicated times.

### Western blot analysis

Protein samples from HeLa or HCT116 cells were prepared in M-PER lysis buffer (Thermo Scientific) supplemented with protease and phosphatase inhibitors (ROCHE). Aliquots of the extracted proteins (approximately 30 μg/lane) were resolved on 12% SDS-PAGE gel (Bio-Rad) and then subjected to immunoblotting using antibodies specific for ACC (mouse monoclonal IgG clone D-5, cat# SC-11427, Santa Cruz Biotechnology), p-ACC Ser79 (rabbit polyclonal IgG clone D7D11, cat# 11818, Cell Signaling Technology), AMPK (mouse monoclonal IgG, cat# 2793, Cell Signaling Technology), p-AMPK (rabbit polyclonal IgG, cat# 2531, Cell Signaling Technology), ATG7 (rabbit polyclonal IgG clone D12B11, cat# 8558, Cell Signaling Technology), Beclin 1 (rabbit polyclonal IgG clone H-300, cat# SC-11427, Santa Cruz Biotechnology), pro-caspase-3 and caspase-3 (rabbit polyclonal IgG, cat# 9665, Cell Signaling Technology), GAPDH (mouse monoclonal IgG, cat# MAB274, Chemicon International), 4EBP1 (rabbit polyclonal IgG, cat# 9452, Cell Signaling Technology), p-4EBP1 Thr37/46 (rabbit polyclonal IgG clone 236B4, cat# 2855, Cell Signaling Technology), LC3 I and II (rabbit polyclonal IgG, cat# 9748, Cell Signaling Technology), p62/SQSTM1 (rabbit polyclonal IgG, cat# NBP1-42822, Novus Biologicals), PC2 (rabbit polyclonal IgG clone H-280, cat# sc-25749, Santa Cruz Biotechnology), PI3K-III/VPS34 (rabbit polyclonal IgG, cat# 3811, Cell Signaling Technology), S6 (mouse monoclonal IgG clone 54D2, cat# 2317, Cell Signaling Technology), p-S6 Ser235/236 (rabbit polyclonal IgG clone 236B4, cat# 2211, Cell Signaling Technology), mTOR (mouse monoclonal IgG clone L27D4, cat# 4517, Cell Signaling Technology), p-mTOR Ser2481 (rabbit polyclonal IgG, cat# , Cell Signaling Technology) and α-tubulin (mouse monoclonal IgG clone DMA, cat# T9026, Sigma-Aldrich). Membranes were then incubated with secondary goat anti-mouse or anti-rabbit IgG conjugated to horseradish peroxidase (SouthernBiotech, Birmingham, AL, USA) prior to revelation using the ECL Detection Kit (Amersham Pharmacia, Pittsburgh, PA, USA). Gels were visualized and quantified with ImageJ software (http://rsb.info.nih.gov/ij/).

### Confocal and fluorescence microscopy

Following treatment, cells were washed twice with ice-cold PBS, fixed in paraformaldehyde (4% w/v) for 15 min, permeabilized with Triton 0.1%, PBS for 10 min and blocked in 3% BSA-PBS for 1 h. Nuclei were counterstained with Hoechst 33342 (10 mg/mL) (Molecular Probes). Fluorescence and confocal fluorescence images were captured using an IRE2 microscope equipped with a DC300F camera (both from Leica Microsystems GmbH, Wetzlar, Germany) and an LSM 510 microscope (Carl Zeiss, Jena, Germany). Images were analyzed with ImageJ software (http://rsb.info.nih.gov/ij/).

### Cytofluorometry

The following fluorochromes were used to assess for apoptosis-associated changes: 3,3′-dihexyloxacarbocyanine iodide (DiOC_6_(3), 40 nM, Molecular Probes, Invitrogen), for quantification of mitochondrial transmembrane potential (∆Ψ_m_), and propidium iodide (PI, 1 μg/ml, Molecular Probes, Invitrogen), to determine cell viability [31]. After trypsinization, cells were stained with DiOC_6_(3) for 30 min at 37°C. Cells were then stained with PI for 5 min at 37°C, followed by cytofluorometric analysis with FACS Vantage equipment (Becton Dickinson, San Jose, CA, USA).

### RNA isolation and RT-qPCR assay

Total RNA was isolated from HeLa cells using Trizol reagent. A total of 100-250 ng RNA was used for reverse transcription with the iScript kit (Bio-Rad). The reverse transcription product was then diluted 10-fold with ddH2O. 2 mL of DNA was used for quantitative PCR analysis (StepOnePlus Real-Time PCR System). The forward and reverse primers for *MAP1LC3B* were CCGTCGGAGAAGACCTTCAA and GCATAGACCATGTACAGGAA, respectively. *GAPDH* was evaluated as a housekeeping gene. The forward and reverse primers were TCAACGACCACTTTGTCAAGCTCA and GCTGGTGGTCCAGGGGTCTTACT, respectively.

### Results and statistical analysis

Results are shown as the mean ± SEM from at least three independent experiments. Statistical analyses were performed using one-way ANOVA (GraphPad Software, Inc.). *p* < 0.05 was considered to be statistically significant.

### Abbreviations

Acetyl-CoA carboxylase (ACC), AMP-dependent kinase (AMPK), A.U. (arbitrary units), Bafilomycin A1 (BafA1), Chinese hamster ovary (CHO), class III phosphatidylinositol 3-kinase complex (PI3KC3), human cervical cancer (HeLa) cells, human colon tumor (HCT116) cells, mechanistic target of rapamycin (mTOR), nuclear factor kappa beta (NF-κB), polycystin-2 (PC2), polycystic kidney disease (PKD), p38 mitogen-activated protein kinase (p38 MAPK), vacuolar protein sorting 34 (VPS34), 4E-binding protein 1 (4EBP1), uncoordinated 51-like kinase 1 protein (ULK1).

## SUPPLEMENTARY MATERIALS FIGURE


