# Supplementary material for: Hyperosmotic stress stimulates autophagy via polycystin-2
Source: Oncotarget. 2017 Jul 5;8(34):55984–97. doi: 10.18632/oncotarget.18995 (PMC5593539; doi:10.18632/oncotarget.18995)
Supplement: Supplementary file 1 [file oncotarget-08-55984-s001.pdf]

Hyperosmotic stress stimulates autophagy via polycystin-2

SUPPLEMENTARY MATERIALS

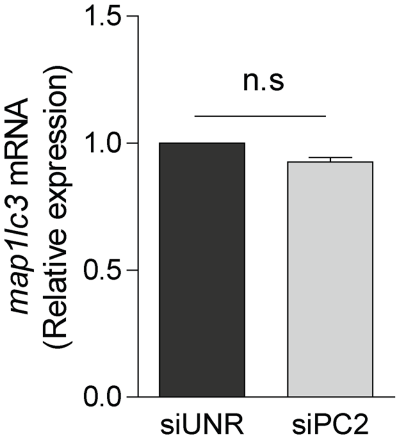

Supplementary Figure 1: PC2 does not regulate MAP1LC3B gene expression
